# Cognitive functioning in children with internalising, externalising and dysregulation problems: a population-based study

**DOI:** 10.1007/s00787-016-0903-9

**Published:** 2016-09-19

**Authors:** Laura M. E. Blanken, Tonya White, Sabine E. Mous, Maartje Basten, Ryan L. Muetzel, Vincent W. V. Jaddoe, Marjolein Wals, Jan van der Ende, Frank C. Verhulst, Henning Tiemeier

**Affiliations:** 1 Department of Child and Adolescent Psychiatry, Erasmus MC-Sophia, Rotterdam, The Netherlands; 2 The Generation R Study Group, Erasmus MC, Rotterdam, The Netherlands; 3 Department of Radiology, Erasmus MC, Rotterdam, The Netherlands; 4 Department of Paediatrics, Erasmus MC-Sophia, Rotterdam, The Netherlands; 5 Department of Epidemiology, Erasmus MC, room Na-2818, P.O.Box 2040, 3000 CA Rotterdam, The Netherlands; 6 Institute of Psychology, Erasmus University, Rotterdam, The Netherlands

**Keywords:** Internalising symptoms, Externalising symptoms, Cognition

## Abstract

Psychiatric symptoms in childhood are closely related to neurocognitive deficits. However, it is unclear whether internalising and externalising symptoms are associated with general or distinct cognitive problems. We examined the relation between different types of psychiatric symptoms and neurocognitive functioning in a population-based sample of 1177 school-aged children. Internalising and externalising behaviour was studied both continuously and categorically. For continuous, variable-centred analyses, broadband scores of internalising and externalising symptoms were used. However, these measures are strongly correlated, which may prevent identification of distinct cognitive patterns. To distinguish groups of children with relatively homogeneous symptom patterns, a latent profile analysis of symptoms at age 6 yielded four exclusive groups of children: a class of children with predominantly internalising symptoms, a class with externalising symptoms, a class with co-occurring internalising and externalising symptoms, that resembles the CBCL dysregulation profile and a class with no problems. Five domains of neurocognitive ability were tested: attention/executive functioning, language, memory and learning, sensorimotor functioning, and visuospatial processing. Consistently, these two different modelling approaches demonstrated that children with internalising and externalising symptoms show distinct cognitive profiles. Children with more externalising symptoms performed lower in the attention/executive functioning domain, while children with more internalising symptoms showed impairment in verbal fluency and memory. In the most severely affected class of children with internalising and externalising symptoms, we found specific impairment in the sensorimotor domain. This study illustrates the specific interrelation of internalising and externalising symptoms and cognition in young children.

**Electronic supplementary material:**

The online version of this article (doi:10.1007/s00787-016-0903-9) contains supplementary material, which is available to authorized users.

## Introduction

In child development, cognitive functioning and psychopathology are closely intertwined. The school-age years are a period of abundant neurodevelopment, characterized by refinement of cognitive skills while, in some children, psychiatric symptoms emerge. Often, a disruption in one area of development is accompanied by impairment in the other, which may reflect a common underlying neurodevelopmental problem [1]. The relation of cognition and psychopathology is particularly well illustrated by developmental disorders, such as ADHD and ASD [2], that are often characterized by lower intelligence or even intellectual impairment. While IQ provides a good measure of general cognitive ability, cognition is a broad construct with various domains, each of which can be selectively impaired or intact. There is increasing attention to assess which specific aspects of cognition are impaired in child psychiatric disorders. Such cognitive impairment can be shared across different disorders, but it may also be distinct for different types of psychopathology. Internalising and externalising disorders are two presentations of psychopathology at a young age, that are thought to emerge from partly distinct pathways, both in terms of genetics [3] and underlying brain correlates [4] and predispose for different types of psychopathology later in life [5, 6]. However, it is less clear whether distinct cognitive patterns exist for internalising and externalising symptoms at a young age.

Cognitive problems in psychopathology have been studied particularly in the context of executive functioning, a broad construct of different abilities to regulate behaviour, such as the ability to pay attention or to inhibit responses. Externalising disorders such as ADHD and disruptive behavioural disorders have been conceptualised as arising from a set of primarily frontally mediated executive function deficits, including attention, planning, working memory and response inhibition [7–11]. There is more debate about the specific deficits in anxiety and mood disorders, that are primarily related to neural circuitry linking limbic structures to frontal regions [12]. Neuropsychological impairment of executive functioning has been reported [13], most notably in visual and working memory in paediatric or adolescent depression [14, 15], while differences in processing speed have also been reported [16]. Attention has also been implicated in paediatric depression [17]. While some childhood anxiety disorders, like OCD in children occur with impairments of executive functioning abilities like mental set-shifting [18], or full-scale IQ [19] they have also been related to impairment in verbal processing [20]. However, it is unclear whether these differences reflect specific lingual processes or aspects of executive functioning, such as impaired attention or working memory. In addition, some studies focused on the neurocognitive implications of co-occurring high levels of internalising and externalising symptoms, for instance in children with ADHD with comorbid internalising symptoms. These studies show inconsistent results, that vary between better test performance [21], no difference [22], to worse performance in tasks of attention, response inhibition and working memory [23] than children with ADHD only.

In general, there is considerable heterogeneity in the literature relating cognition to child psychopathology and studies in young children are relatively scarce. Yet, it is especially important to study younger children, as patterns of emerging psychopathology and impaired cognition can provide more insight in the aetiology relatively unobscured by chronicity of symptoms or treatment effects. Further, the close relation between cognition and psychopathology in young age provides a potentially powerful treatment target for early intervention. So far, many clinical studies tend to focus on a limited range of cognitive domains within small samples of children that have one or more clinically diagnosed psychiatric disorders. Importantly, within this framework, the question of specificity of cognitive impairments cannot be answered by design. Child psychopathology is characterised by a high level of comorbidity between different symptom types, crossing the boundaries of diagnoses. Children that have a high level of externalising symptoms, such as aggression tend to also have internalising symptoms, such as anxiety or depressed mood [24]. Knowledge about specific patterns of cognitive impairment per symptom type could point to specific genetic or neurobiological pathways [8] and help in targeted treatment decisions and predictions of the clinical course of specific symptoms.

An alternative to the case–control framework is provided by studying psychopathology on the symptom level, focusing on continuous trait phenotypes [25]. However, various continuously measured psychiatric symptoms are also inter-related. Although the associated cognitive problems may in fact be distinct for each symptom type, the strong correlation between internalising and externalising symptoms can obscure any potential specificity of associations. Therefore, in the current study, we also used a different approach to address the relation of internalising and externalising symptoms and cognition that added an element of specificity. To this aim, we complemented the traditional variable-based approach with a person-centred approach that allows to distinguish between different symptom profiles in case of heterogeneity [26]. Previously, we applied a latent profile analysis (LPA) to quantitative behavioural and emotional symptom data of more than 6,000 children to identify four broad, but exclusive classes with different patterns of symptoms [27]. Three of these classes included children with problem behaviour: a class of children with predominantly externalising symptoms, a class with predominantly internalising symptoms and a small class with both internalising and externalising symptoms that bears a resemblance to the CBCL Dysregulation Profile, a phenotype of high comorbidity that is associated with a broad range of later psychopathology [28]. In this population-based cohort, the majority of children belonged to a class without psychopathology. The three classes of problem behaviour have so far only been related to a general, global measure of non-verbal intelligence [29]. Here, we aimed to further specify these differences by assessing more specific cognitive sub-domains using an extensive neuropsychological test battery that covers five domains of neurocognitive ability: attention/executive functioning, language, memory, sensorimotor functioning and visuospatial ability.

In the current study, we assessed the relation between cognition and psychiatric symptoms in more than 1,000 school-aged children. In line with recommendations of the RDoC initiative, we used continuous measures of internalising and externalising symptoms to capture the full spectrum of symptom severity, including subclinical symptoms [30]. However, these measures were strongly correlated, so to identify unique patterns of impairment across different symptom types, we used the previously identified problem classes representing more homogeneous groups in terms of symptomatology. Based on the literature, we hypothesised that children with externalising symptoms show poorer performance on the attention/executive functioning domain. Further, we hypothesised that children with internalising symptoms would show moderately impaired test performance in the domains of language, memory and attention. In the class of children with high levels of both internalising and externalising symptoms, we expected widespread impairment, since they likely reflect the most severely affected group.

Additionally, we tested if any impairments were independent of demographic and maternal factors or autistic symptoms. Finally, we explored whether any observed differences reflect global cognitive impairment or more specific deficits by adjusting for IQ.

## Methods

### Participants

This study included a subgroup of children from the Generation R Study, a multi-ethnic population-based cohort, investigating children’s health, growth and development from foetal life onwards in Rotterdam, the Netherlands. An overview of the Generation R Study design and population is provided elsewhere [31].

As part of a previously described sub-study [32], 1307 participants completed a neuropsychological test battery. In this sub-study, children with specific traits (including autistic traits and externalising disorders, were oversampled (see Supplementary Figure 1 for a consort diagram). Oversampling of children with problem behaviour increased the variability, which improved power of the analyses and helped in achieving a more normal distribution of psychopathology symptoms, which are generally strongly right skewed in the general population.

One hundred thirty children had missing information on problem behaviour and were excluded, resulting in a study sample of 1177 children.

The study was approved by the Medical Ethics Committee (METC) of the Erasmus Medical Centre. Written informed consent was obtained from the parents of all participants.

### Internalising and externalising symptoms

When the children were approximately 6 years of age, mothers of 6,131 children completed the Child Behaviour Checklist (CBCL/1.5–5). The CBCL is a widely used instrument has been shown to have good reliability and validity [33] and is generalizable across 23 societies [34]. It measures childhood psychiatric symptoms quantitatively; both in the clinical and non-clinical range and thereby captures the full range of severity. It contains internally consistent Internalizing and Externalizing broadband scales that globally correspond to mood and anxiety disorders and disruptive behaviour disorders, respectively [6]. The Internalizing and Externalizing broadband scales are able to measure broad behavioural constructs in early childhood that have been shown to predict later, more specific psychopathology [35, 36]. The Internalising scale consists of the following four scales: Emotionally Reactive; Anxious/Depressed; Somatic Complaints; and Withdrawn. The Externalising scale contains two scales: Attention Problems and Aggressive Behaviour [27]. In our first approach, we related the continuous broadband scores to cognitive functioning. Second, to explore specific cognitive problems of internalising and externalising symptoms, we defined four classes of children with distinct patterns of behavioural and emotional symptoms that were obtained by a latent profile analysis performed on T-scores of CBCL syndrome scales that constitute the internalising and externalising broadband scales. These included a class of children without problems, a class with predominantly internalising symptoms; a class with externalising symptoms and emotional reactivity, further referred to as ‘externalising’; and a class with high scores on both the internalising and externalising scales. This class is referred to as the dysregulation class. Details on the full modelling strategy and fit indices of models including 1 to 5 classes are described by Basten et al. [27]. The model with four classes provided good fit measures, and the most meaningful distinction of qualitatively different profiles.

The most likely class memberships derived from this analysis were used in this study (see Table 1 for percentages). This was justified by the high entropy (0.98) of the latent class model [27, 37]. The intrinsic relation of internalising and externalising symptoms, and scores of children in the four classes on these broadband scales are illustrated in Supplementary Figure 2.

**Table 1:** Participant characteristics (*n* = 1177)

	Dysregulation	Internalizing problems	Externalizing problems	No problems group	*p* value
	*n* = 63	*n* = 105	*n* = 171	*n* = 838	
Child characteristics					
Gender (% boy)	65.1	46.7	65.5	51.3	0.001
Ethnicity (%)					
Dutch	49.2	54.3	60.2	74.0	<0.001
Other Western	9.5	5.7	8.2	7.9	
Non-Western	41.3	40.0	31.6	18.1	
Age at CBCL (years)	6.0 (0.4)	6.0 (0.4)	6.0 (0.3)	6.0 (0.4)	0.812
Range	5.0–7.9	5.3–7.7	5.3–7.4	4.9–7.9	
Age at NEPSY-II NL (years)	7.6 (0.9)	8.0 (1.0)	8.0 (1.1)	7.9 (1.0)	0.017
Range	6.3–9.6	6.1–10.7	6.1–10.7	6.1–10.4	
IQ (non verbal)	95.3 (15.0)	99.0 (14.1)	98.9 (15.4)	103.2 (14.0)	<0.001
Range	67–135	61–127	50–135	50–142	
Maternal characteristics					
Monthly household income (%)					<0.001
High	60.3	59.0	75.2	79.5	
Medium	21.0	27.0	14.9	15.4	
Low	17.7	14.0	9.9	5.1	
Alcohol use during pregnancy (%)					0.174
Never	31.0	45.5	41.1	35.4	
Until pregnancy was known	15.5	11.4	16.4	14.4	
Continued occasionally	46.6	33.0	37.7	38.4	
Continued frequently	6.9	10.2	4.8	11.8	
Smoking during pregnancy (%)					0.054
Never	65	76.6	69.0	78.0	
Until pregnancy was known	6.7	8.5	7.7	6.4	
Continued	28.3	14.9	23.2	15.5	

Values are mean and SD unless otherwise indicated

Importantly, the classes were not based on symptom severity thresholds. However, for the interpretation of the profiles, mean T-scores are provided (Supplementary Figure 3).

### Cognitive functioning

Cognitive functioning was measured using the NEPSY-II-NL, an official and validated Dutch translation and adaptation of the North American NEPSY-II battery, that can be used to assess neuropsychological functioning in 5- to 12-year-old children [38]. Tasks are categorized to cover several theoretically derived domains of cognition, including attention/executive functioning, language, memory and learning, sensorimotor functioning, and visuospatial processing. The task battery is sensitive to inter-individual differences, not only in clinical groups but also in the general population [39]. Acceptable to good reliability and validity have been reported for the NEPSY-II [40]. Due to time constraints, a selection of tests from the NEPSY was chosen such that five areas of cognitive ability were measured: attention/executive functioning, language, memory and learning, sensorimotor functioning, and visuospatial processing. The battery was administered by trained research assistants and took approximately 55 min.

As the NEPSY-II-NL does not provide domain-specific summary scores, a data reduction technique was used to derive them empirically. Summary scores for four NEPSY-II-NL test domains (attention/executive functioning, language, memory and learning, and visuospatial processing) were derived using a principal component analysis (PCA) on all test scores belonging to that domain. The first unrotated factor score was selected as the summary score for each cognitive domain. For the sensorimotor domain, this procedure was slightly different, as described below. The different subtest scores that contributed to each domain score are described in the Supplementary material. In Supplementary Table 1, the correlation with the corresponding domain scores that they contributed to is provided.

The term ‘cognitive problems’ refers to the continuum of problems that a child may have and does not imply a severity threshold.

### Selected tasks for each of the domains of the NEPSY-II-NL

#### Attention and executive functioning

The first task of this domain was the Auditory Attention and Response Set Task. In the Auditory Attention component of this task the children were presented recordings of words and asked to selectively respond to the word ‘Red’ by touching the red circle on the sheet in front of them. The sheet also contains a blue, black, yellow and red circle, but these had to be ignored, as well as all non-colour words. Touching the right circle within 2 s indicates a correct response.

Following the Auditory Attention component, Response Set was performed, which taps into response inhibition and working memory. In this task, children are asked to respond to the word ‘Red’ by touching the yellow circle, respond to ‘Yellow’ by touching the red circle and lastly, respond to the word ‘Blue’ by touching the blue circle. All the other colours should be ignored. Touching the right circle within 2 s equals a correct response, whereas touching another circle or a delayed response (>2 s) are incorrect. Performance in both components of the Auditory Attention and Response Set task was measured using four summary scores per component: the total score of correct responses and the total number of commission, omission, and inhibition errors. Omission errors indicate that the child failed to respond. Commission errors are delayed or incorrect responses. Inhibitory errors occur when the child responds to a colour word when no response was warranted.

The second task in this domain is the Statue task. This task requires a child to maintain a ‘statue-like’ body position for a period of 75 s, while ignoring environmental distractors. Summary measures from the Statue task include the total number of body movements, eye openings, sound productions, and a total score.

#### Language

The language skills domain involves a test of verbal fluency, the Word Generation task. This task measures how many words a child can generate within 60 s in two semantic categories: animals and food or drinks. The total semantic score is the sum of the total number of unique, existing words for both categories.

#### Memory and learning

The memory and learning domain entailed the memory for faces task, with an immediate and delayed memory component. During this task the child is first presented multiple series of three faces, after which the child has to identify the face it has previously seen, out of another series of three faces. The delayed recall component of this task was assessed after a delay period of 15–25 min. A total correct score was calculated for both the immediate and delayed recall.

The verbal memory task that we assessed is the Narrative Memory task. This task measures immediate free recall, cued recall, and (passive) recognition of verbal information. In this task, children were presented a short story after which they were asked to provide as many details as they could remember. Subsequently, children were asked specific questions about the story (cued recall), and finally questions that only required yes and no answers were provided (recognition). The Narrative Memory task provides a total correct score for the free and cued recall combined the free recall only, and for recognition.

#### Sensorimotor functioning

In the paper-and-pencil task Visuomotor Precision, the child is asked to draw a line as quickly and as accurately as possible in between the boundaries of a paper path. For this task, two separate scores were derived. Due to the fact that different summary scores in this task may reflect distinct strategies (e.g., fast with many errors vs. slow but more accurate), it was not possible to derive a single meaningful sensorimotor factor out of the separate scores. Therefore, two independent scores were derived. The primary sensorimotor score is a speed-accuracy trade-off score, based on the product of the standardized time and number of errors in this task, while the secondary sensorimotor score is based on the number of compensatory pencil lifts while performing the task.

#### Visuospatial processing

The visuospatial processing domain consisted of three different tasks. The Arrows task measures the child’s ability to judge the direction of an arrow by asking the child to select the arrow(s) that point(s) to centre of a target from a set of arrows. The summary score for the Arrows task is the total number of correct responses. The Geometric Puzzles task measures mental rotation, visuospatial working memory, and attention to detail. This task requires a child to discriminate which abstract figures in a set match those within a grid containing multiple abstract figures. Figures in the grid are often rotated and thus appear different than the example figure. Finally, the Route Finding task was administered, which measures visuospatial relations, orientation, and direction. The child uses a skeleton map of a specific route to translate this route onto another map. The summary score obtained from this task is the total correct score from a series of 10 maps.

### Covariates

Several covariates were considered, based on previously described associations of sociodemographic factors and prenatal exposures with child psychopathology and cognitive functioning [41–43]. Intelligence was not taken into account as a default covariate, as this carries the risk of overadjustment in the context of developmental psychopathology [42]. However, we explored whether the differences reflected global or specific cognitive deficits, by additionally correcting the fully adjusted analyses for non-verbal IQ. Non-verbal intelligence of the child was assessed at approximately 6 years of age using two subtests of the Snijders-Oomen Niet-verbale intelligentie test–revisie (SON-R 2.5–7), a nonverbal intelligence test suited for children between 2.5–7 years of age [44]: Mosaics (which assesses spatial visualization abilities), and Categories (which assesses abstract reasoning abilities). Raw scores from these two subtests were standardized to reflect a mean and standard deviation of the Dutch norm population age 2½–7 years and subsequently converted into SON-R IQ score using age-specific reference scores provided in the SON-R 2½–7 manual (mean = 100, SD = 15). Child ethnicity was defined according to the ethnicity categorisation of Statistics Netherlands [45]. Children with both parents born in the Netherlands were considered Dutch and children were classified as non-Dutch (further categorised as ‘other Western’ or ‘other non-Western’) if one parent was born outside the Netherlands. Household income was defined by the total net monthly income of the household and categorised into three categories: income below <1200 Euros per month (below social security level), 1200–2200 Euros (low income), and >2200 Euros (modal income and above). Prenatal smoking and alcohol use were categorised into ‘No’, ‘Until pregnancy was known’ and ‘Continued during pregnancy’, based on the information of repeated questionnaires during pregnancy. For continued alcohol use, there were two categories: ‘continued occasionally’ and ‘continued frequently’. Autistic traits were assessed using the 18-item short form of the social responsiveness scale (SRS), a parent-reported questionnaire about the child’s social behaviour during the past 6 months. The Social Responsiveness Scale provides a quantitative measure of autistic traits. The authors recommend cut-offs for screening in population-based settings (consistent with weighted Social Responsiveness Scale scores of 1.078 for boys 1.000 for girls) [46].

### Data analysis

To examine the relation between internalising and externalising symptoms and cognition, we performed linear regression analyses using the NEPSY-II-NL domain scores as the dependent variable. In our first approach, the CBCL broadband scales were used as the independent variable in two separate regression analyses. Scores that showed moderate negative skew were square root transformed to approach normality. In a second approach, most likely class membership was used as the independent variable. Class membership was dummy coded, with the no problems class as the reference.

All analyses were adjusted for gender and age at the time of the CBCL/1.5-5, as well as age at time of the NEPSY-II NL. In a second model, other variables were included as covariates if they changed the effect estimate (unstandardised regression coefficient B) by 5 % or more.

Missing values of covariates (max 13.4 %) were imputed. We computed five imputed datasets.

While IQ was not a default covariate, a second set of regression analyses was performed after adding it as a covariate, to distinguish between a global intellectual problem or impairment of a specific neurocognitive domain.

A sensitivity analysis was conducted to further examine the association between the classes and NEPSY-II-NL performance. We excluded those that screened positive on a questionnaire of autistic symptoms, to study whether class differences in autistic traits explained the observed results. In the classes resulting from our latent profile analysis, there is not one class that specifically identifies children with ASD traits (although they are likely overrepresented in the dysregulation class). Further, autistic traits are quite common in our population-based sample, as children with autistic symptoms were specifically targeted in the recruitment for this sub-study (see Supplementary Figure 1 and [32]).

All analyses were conducted using SPSS (IBM SPSS Statistics version 21.0).

## Results

### Participant characteristics

Child and maternal characteristics for each of the four classes are presented in Table 1. As expected, the dysregulation and externalising classes included the highest proportion of boys. Non-verbal intelligence levels were lower in the problem classes if compared to the reference class, in line with earlier reported differences [29]. The dysregulation class scored 8.0 points lower on non-verbal IQ (95 % CI: −11.9; −4.0, *p* < 0.001) than the reference class, while the internalising and externalising classes showed 4.2 and 4.4 points lower, respectively (95 % CI: −7.3; −1.1, *p* = 0.008 and 95 % CI: −6.9; −1.9, adjusted for age and gender). A non-response analysis is presented in the Supplement. The latent class approach distinguishes children with little symptoms from children with mostly internalising or mostly externalising symptoms, while in the dysregulation class, no meaningful distinction can be made between the two symptom types (Supplementary Figure 2).

Due to oversampling of children with specific traits (including autistic traits and externalising disorders, see Supplementary Figure 1 for a consort diagram), the prevalence of each of the problem classes was higher than in the original, larger sample [27]. In this sample, 8.9 % of children were part of the internalising class, 14.5 % were part of the externalising class, 5.4 % belonged to the dysregulation class and the other children (71.2 %) were part of the reference class.

### CBCL broadband scales

As expected, the correlation between internalising and externalising symptoms was strong [*r*(1175) = .73, *p* < 0.001]. Associations between the CBCL internalising and externalising broadband scales with cognitive domains are presented in Supplementary Table 2. After adjustment for confounders, internalising symptoms were associated with lower performance in the domains of attention/executive functioning, language and memory and learning (*B* = −0.11, 95 % CI (−0.12; −0.03], *p* = 0.001, *B* = −0.06, 95 % CI [−0.10, −0.03], *p* = 0.001) and (*B* = −0.07, 95 % CI [−0.11, −0.03], *p* < 0.001), respectively). Externalising symptoms were associated with lower scores in attention/executive functioning, as well as secondary sensorimotor domain scores after adjustment for confounders (*B* = −0.07, 95 % CI [−0.11, −0.03], *p* < 0.001) and (*B* = −0.05, 95 % CI [−0.09, −0.01], *p* = 0.02), respectively.

### Cognitive functioning across the four classes

**Fig. 1:**
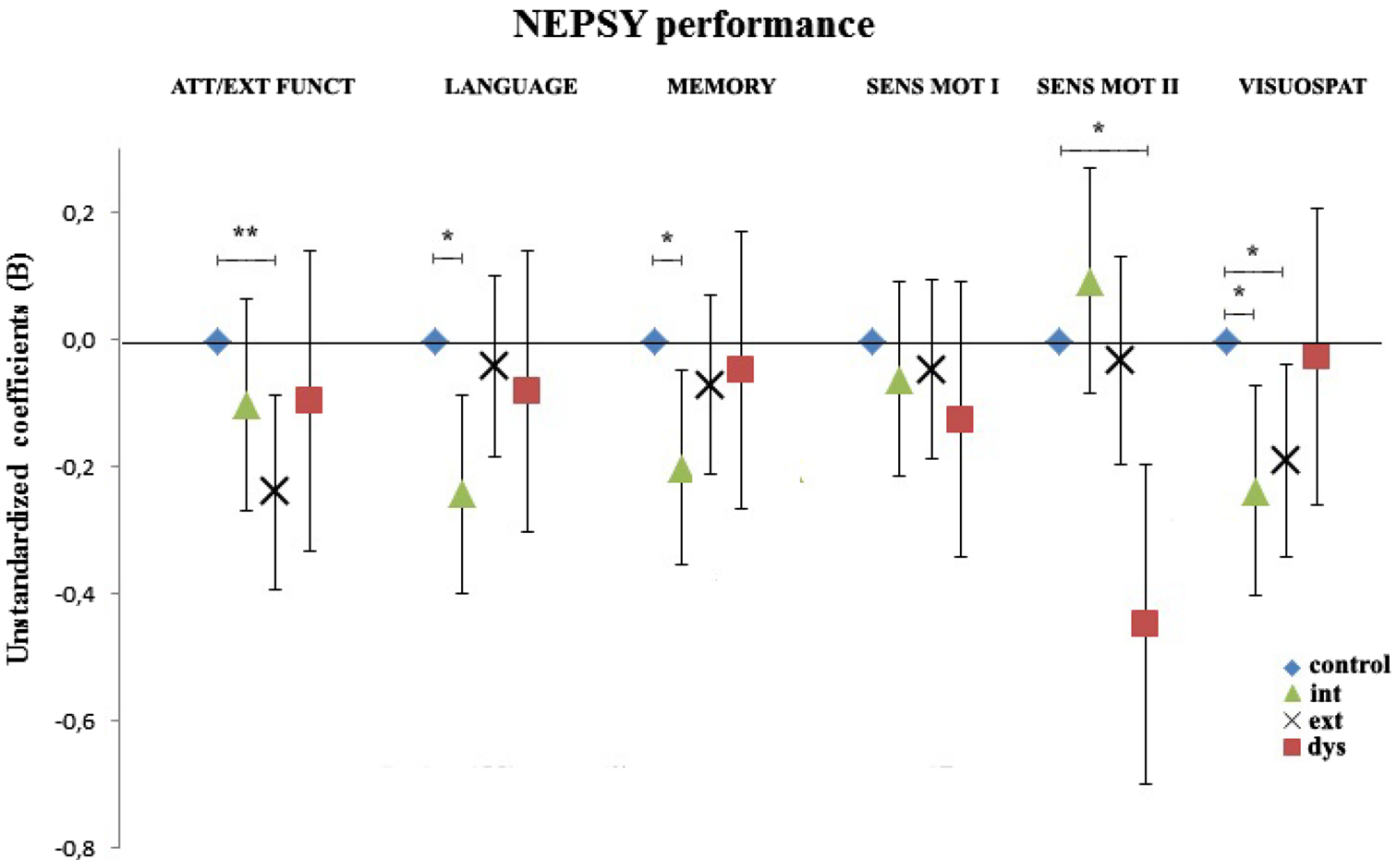
Associations between the internalising class, the externalising class and the dysregulation class and performance on domains of the NEPSY-II-NL (*n* = 1177). The no problems class (*n* = 838) is the reference. There were 171 children in the externalising class, 105 children in the internalising class and 63 children in the dysregulation class. Regression model was adjusted for gender, age at the time of the CBCL/1.5-5, and age at the NEPSY-II NL. *p < 0.01, **p < 0.001. *Error bars* represent 95 % confidence intervals of the regression coefficients. The no problems class is the reference and has no *error bars*

To distinguish distinct cognitive problems of internalising, externalising and dysregulation symptoms, we compared performance of children in the problem classes to the reference class in all neuropsychological subdomains (Fig. 1). In line with most other studies, analyses are adjusted for age and gender only. Children in the externalising class scored lower than the reference class in the attention/executive functioning domain (*B* = −0.28, 95 % CI [−0.43, −0.12], *p* < 0.001) and in the visuospatial domain (*B* = −0.23, 95 % CI [−0.38, −0.08], *p* = 0.003). Contrary to the broadband score approach, children with externalising symptoms did not have lower secondary sensorimotor domain scores.

Children in the internalising class performed worse in the language and memory domains (*B* = −0.30, 95 % CI [−0.47, −0.12], *p* = 0.001 and *B* = −0.25, 95 % CI [−0.42, −0.08], *p* = 0.004). Children in the internalising class also performed worse in the visuospatial domain (*B* = −0.29, 95 % CI [−0.48, −0.11], *p* = 0.002). In contrast to the broadband score approach, children with internalising symptoms did not show lower performance in the attention/executive functioning domain. Children in the dysregulation class had lower secondary sensorimotor domain scores compared to the control class (*B* = −0.48, 95 % CI [−0.74, −0.23], *p* = <0.001), while there were no differences in the performance scores in other domains. Generally, children in the dysregulation class had the greatest SD-scores (data not shown).

In a second model, we explored if the differences in performance withstood additional adjustment for demographic and maternal factors. Fully adjusted models are presented in Table 2 (adjusted for age, gender, ethnicity, income, alcohol and smoking during pregnancy). The patterns of performance remained similar. However, there were no longer any differences in visuospatial functioning between either the internalising or the externalising classes and the reference class. Children in the internalising class showed lower performance in the language and memory domains.

**Table 2:** Association between the dysregulation profile, the internalising profile and the externalising profiles and performance on domains of the NEPSY-II NL (*n* = 1177)

	Internalising	*p*	Externalising	*p*	Dysregulation	*p*
	*n* = 105		*n* = 171		*n* = 63	
	*B* (95 % CI)		*B* (95 % CI)		*B* (95 % CI)	
Attention and executive functioning	–0.10 (–0.29; 0.19)	0.300	–0.25 (–0.40; –0.09)	**0.002**	–0.10 (–0.34; 0.14)	0.417
Language	–0.19 (–0.36; –0.01)	**0.035**	–0.02 (–0.16; 0.12)	0.755	–0.02 (–0.23; 0.20)	0.893
Memory and Learning	–0.19 (–0.37; –0.02)	**0.029**	–0.07 (–0.21; 0.07)	0.347	–0.06 (–0.27; 0.16)	0.607
Sensorimotor, primary score	–0.06 (–0.24; 0.11)	0.482	–0.06 (–0.21; 0.08)	0.378	–0.14 (–0.36; 0.08)	0.210
Sensorimotor, secondary score	0.09 (–0.11; 0.30)	0.365	–0.07 (–0.23; 0.10)	0.419	–0.48 (–0.73; –0.22)	**<0.001**
Visuospatial	–0.15 (–0.33; 0.03)	0.104	–0.12 (–0.27; 0.03)	0.113	0.06 (–0.17; 0.29)	0.601

The no problems group (*n* = 838) is the reference. The model was adjusted for age at CBCL and age at NEPSY-II-NL, gender, ethnicity, household income, alcohol and smoking during pregnancy

Significant *p* values are in bold

We explored whether the differences reflected global or specific cognitive deficits, by additionally correcting the analyses for non-verbal intelligence. In these most stringent analyses, major differences remained. The externalising class had lower performance in attention/executive functioning and the dysregulation class had a lower secondary sensorimotor domain score (pencil lifts) domain (*B* = −0.23, 95 % CI [−0.38, −0.07], *p* = 0.004 and *B* = −0.48, 95 % CI [−0.73, −0.23], *p* = <0.001, respectively).

The internalising class showed poorer performance in the language and memory domains, independent of IQ (*B* = −0.18, 95 % CI [−0.35, −0.00], *p* = 0.048 and *B* = −0.18, 95 % CI [−0.35, −0.01], *p* = 0.043).

### Sensitivity analysis excluding children with ASD

In a sensitivity analysis, we explored whether the differences in performance were dependent on the presence of a probable diagnosis of ASD. In 86.0 % of this sample the Social Responsiveness Scale was available. After excluding the children with possible ASD (*n* = 35) and children without information on autistic traits (*n* = 164), we observed a very similar pattern of cognitive performance (Supplementary Figure 4), indicating that autistic traits did not explain the results. Similarly, a second sensitivity analysis excluding children with a non-verbal intelligence below 70 revealed the same patterns of differences.

## Discussion

In this study, we found a relation between neurocognitive impairment and internalising and externalising symptoms in children from the general population. Despite the high overlap between continuous internalising and externalising symptom scores, we were able to test the specificity of the associations, using empirically derived and exclusive classes based on the children’s behavioural and emotional symptoms. These included a class with predominantly internalising symptoms; a class with externalising symptoms; and a separate class with high scores on both the internalising and externalising scales, which we labelled a dysregulation class. Our approach of evaluating both continuous and categorical measures revealed distinct patterns of cognitive impairment in children with predominantly internalising and externalising symptoms. Both approaches yielded comparable neurocognitive patterns. However, the class approach can help disentangle the specific cognitive problems of each symptom group by adjusting (without problems of collinearity). The specific relation of the externalising class with performance in the attention/executive functioning domain clearly illustrates this. Further, the main findings were independent of IQ, which indicates a specific relation of internalising and externalising symptoms and these domains, independent of general cognitive ability.

We found that children with mostly externalising symptoms showed impairment of the attention/executive functioning domain only. This difference remained after adjustment for a global measure of intelligence, indicating a specific relation between behaviour and cognitive impairment, unrelated to global intelligence. This is in line with findings of specific cognitive impairment in ADHD and disruptive behavioural disorders, notably in the domain of executive function [7], although global impairment has also been reported [47]. Our finding is consistent with an influential theory of ADHD, that states that both the cognitive and the behavioural aspects of it reflect a core impairment in inhibitory control. This leads to lower ability to internally regulate behaviour [48]. Internal regulation and executive functions like sustained attention are thought to be modulated by the prefrontal cortex and its striatal and parietal connections. Altered connectivity in networks involving frontal regions is thought to be central to the neurobiology of ADHD [49].

In contrast, children with mostly internalising symptoms showed impairment in verbal fluency and memory. These processes have been shown to be interrelated. Formation of memories is partly mediated by verbal processes. Next to a large active vocabulary, it is essential to be able to remember new words [50]. Also, in learning disabilities such as Specific Language Disorder or dyslexia, impairments of both aspects have been reported [51]. Unsurprisingly, children with such learning disabilities also tend to have internalising symptoms [52]. However, since we only measured verbal fluency in our study, it is unclear whether the observed impairment of word generation in children with internalising symptoms would be observed in other areas of language. In addition, it is also possible that it reflects an impairment in aspects of executive functioning, as has been suggested by previous reports [53, 54]. In this study, we did not find evidence for impaired executive functioning in children with internalising problems; moreover, the results remained after adjusting for IQ. This indicates that our findings may be specific for verbal processes.

There are several possible neurobiological mechanisms that could underlie these specific cognitive impairments in children with internalising symptoms. Early internalising symptoms have been characterized by disruptions in development of brain regions implicated in memory, such as the hippocampus [55]. Disruption of the HPA-axis, with prolonged overproduction of glucocorticoids causing damage to hippocampal neurons, has been proposed to underlie memory deficits in paediatric depression [56, 57]. Another mechanism thought to underlie both anxiety and depression is suboptimal cortical regulation of the limbic system, including the amygdala and the insula [58]. Altered functioning of such cortical regulatory regions could potentially also affect memory deficits. For instance, a study of major depressive disorder found memory dysfunction to be related to blood flow in the prefrontal cortex and anterior cingulate cortex [59]. Another potential neurobiological pathway is provided in the parietal-frontal integration theory, that poses that connectivity in a network involving parietal and frontal regions is crucial for intelligence and abilities like working memory [60, 61]. It is possible that connectivity in such regions is impaired in children with internalising symptoms. This also meshes well with the decreased word production that we observed in these children, as verbal fluency is associated with activity of several frontal areas, including the left inferior frontal gyrus and the dorsolateral prefrontal cortex [62].

Decreased performance in language tasks has also been reported previously in anxiety disorders [20]. Children in the internalising class scored high on the scales of anxiety and depression, but also on withdrawn behaviour. This constellation of symptoms may reflect a phenotype of more ‘inhibited’ or shy behaviour. Inhibited children are less inclined to talk and have lower scores on tasks that require their spontaneous verbal response, such as the ‘word generation’ task in the NEPSY-II-NL. If the suboptimal performance of these children is a result of their shyness, or general task anxiety, these results are an indicator of their emotional symptoms. Alternatively, their emotional symptoms may limit their social interaction and impede with the development of highly training-dependent cognitive abilities such as language. Another mechanism proposed for lower cognitive performance is that these children are mentally ‘occupied’ by other cognitive processes such as extensive worrying that may engage their working memory [19]. Problems in sustained attention and disruption of the resting state functional MRI attention network have also been reported in children with anxiety and depression [63] and could underlie poorer test performance, and disrupt acquisition and consolidation of new information that is necessary for learning. Although internalising broadband scores were associated with lower attention domain scores, this was likely a result of overlap with externalising symptoms. We did not find this relation using the class approach, which indicates that this relation was likely due to confounding by comorbid externalising symptoms. Likewise, a study in children with anxiety disorders showed that inattention did not mediate the relation between anxiety disorders and intellectual ability [19]. Of note, internalising problems in young children may not represent the same construct as internalising problems at a later age. For instance, the prevalence of depression in such young children is thought to be extremely low, and any internalising problems, especially in the general population, may primarily reflect anxiety and withdrawn behaviour. However, internalising problems at this age are very predictive of internalising disorders at a later age [5].

In children with co-occurring internalising and externalising symptoms, we expected to observe widespread impairment. Children in this highly problematic class showed problems across a variety of scales and resemble the CBCL Dysregulation profile [28]. In a previous study, we found that these children had an 11 point lower nonverbal intelligence score than those without problems [29]. However, contrary to our hypothesis, we did not observe widespread impairment. Possibly, the heterogeneity of the behavioural symptoms is reflected in the neurocognitive profiles. These children had the highest variability in performance across the domains (see SD). Children in this smallest class (*n* = 63) had higher mean performance scores than the internalising and externalising classes, which suggests that at least some children performed above our expectation. Decoupling of intelligence and other aspects of neurocognitive performance has been reported before [64]. Further, selection effects could have occurred in our study. The dysregulation class showed slightly less impaired non-verbal intelligence than previously reported (8 versus 11 points difference). Additionally, there is some evidence that children with ADHD and comorbid anxiety perform better in some cognitive tasks than children with only ADHD [21, 23]. Interestingly, in the sensorimotor domain, we observed impairment. Children with dysregulation lifted their pencil more often while quickly drawing lines through different tracks. This may indicate more compensatory movements. Possibly, this group comprised children with high-functioning ASD. Often, children with ASD show rather peculiar cognitive patterns, with relatively more severe sensorimotor impairment, compared to other cognitive domains [65]. Thus, we performed a sensitivity analysis excluding children that scored above the population screening threshold on an ASD questionnaire After exclusion of these children, associations were attenuated but the differences in IQ between the dysregulation class and the reference class (data not shown) remained. This suggests that children with characteristics of ASD were partly responsible for the low IQ scores attributed to the dysregulation class in our previous study [66].

However, even after excluding these autistic-like children from the current analyses, the sensorimotor differences remained. It cannot be ruled out that the remaining children in the dysregulation class also had syndromes with motor clumsiness as a central feature, such as developmental coordination disorder [65].

The present study has several strengths. We used a large population-based sample and distinguished children with internalising and externalising symptoms using an empirical person-centred classification based on a broad range of behavioural and emotional symptoms. Importantly, our non-problematic class was representative of the general population in the sense that it was not restricted to children without any symptoms at all. The problem classes were not dependent on clinical cut-offs or DSM-criteria. Rather, these classes captured patterns of commonly co-occurring symptoms that reflect the true heterogeneity in child psychopathology. Additionally, information on potential confounding factors was available. Adjusting associations with neuropsychological performance for IQ can be helpful in elucidating specific relations between psychopathology and neurocognitive domains. Further, our neurocognitive test battery encompassed five different neurocognitive domains and provided a broad observational measure of cognition.

This study also has some limitations. First, our sample showed a slight tendency to more privileged families, so we are likely missing children with a higher risk for both cognitive problems and psychopathology. We can only carefully speculate that if the relation between cognition and psychopathology is particularly prominent in these children, as is suggested from clinical studies, our result could represent an underestimation of the true effects.

Second, there was a delay between identification of the behavioural classes and the neuropsychological testing (mean delay 1.85 years). However, correlations between repeated measures of neurocognitive performance during childhood development have been shown to be substantial [67]. In addition, internalising and externalising symptoms at young age correlate strongly with later symptoms [24]. Another limitation is the relatively small sample of children with dysregulation (*n* = 63), although this is unsurprising considering the population-based nature of the study. While there may be subgroups within this class, our study is not powered to explore those. Further, due to the cross-sectional nature of this study, we cannot infer the causal direction of these associations. Additionally, although our task battery measures a wide range of domains, it does not capture all the different constructs of each cognitive domain. This was not feasible for reasons of time and subject burden. For instance, the language domain measures verbal fluency, but does not capture other expressive and receptive aspects of language. Finally, adding measures of cognitive functioning to the latent class analyses could have added to the descriptive validity of the classes. However, this would make the classes less generalizable and cognition could not be tested as a correlate anymore, but would be an intrinsic part of the classification.

In conclusion, the current study shows specific relations between internalising and externalising symptoms and cognitive impairment. First, this information facilitates a better understanding of the underlying aetiology. Internalising symptoms may share neurobiological pathways with verbal fluency and memory impairments, while externalising symptoms appear to be more specifically related to attention and executive functioning. Second, knowledge of the specific cognitive implications of psychopathological symptoms can help clinicians characterise the range of symptoms of an individual child. This is essential in determining the prognosis. Third, our results can potentially help in making informed treatment decisions, particularly if the clinical picture is characterised by comorbidity. For example, to specifically target executive functioning problems in a child that presents with a mixture of symptoms, it is helpful to treat the externalising component of the psychopathology. Finally, a better understanding of cognitive endophenotypes could help identify novel targets for therapeutic intervention.

## Electronic supplementary material

Below is the link to the electronic supplementary material.

Supplementary material 1 (DOCX 191 kb)
