# Supplementary material for: Cognitive functioning in children with internalising, externalising and dysregulation problems: a population-based study
Source: Eur Child Adolesc Psychiatry. 2016 Sep 19;26(4):445–56. doi: 10.1007/s00787-016-0903-9 (PMC5364260; doi:10.1007/s00787-016-0903-9)
Supplement: Supplementary file 1 — Supplementary material 1 (DOCX 191 kb) [file 787_2016_903_MOESM1_ESM.docx]

Cognitive functioning in children with internalising, externalising and dysregulation problems: a population-based study

**Laura M.E. Blanken, MD, MSc^1,2^, Tonya White, MD, PhD^1,3^, Sabine E. Mous, MSc^1,2^, Maartje Basten, PhD^1,2^, Ryan L. Muetzel, MSc^1,2^, Vincent W.V. Jaddoe, MD, PhD^2,4,5^, Marjolein Wals, PhD^1,6^,** **Jan van der Ende, Msc^1^, Frank C. Verhulst, MD, PhD^1^, Henning Tiemeier, MD, PhD^1,5§^**

*Erasmus MC – Sophia, Rotterdam: ^1^Department of Child and Adolescent Psychiatry, ^2^The Generation R Study Group, ^3^Department of Radiology, ^4^Department of Paediatrics, ^5^Department of Epidemiology, ^6^Institute of Psychology, Erasmus University*

^§^Correspondence to: Henning Tiemeier, Erasmus MC, Department of Epidemiology, room Na-2818, P.O.Box 2040, 3000 CA Rotterdam, The Netherlands. Phone: (+31) 10 7043489. E-mail: h.tiemeier@erasmusmc.nl

**Supplementary Fig. 1**Consort diagram.

**Supplementary Fig. 2**

Internalising and externalising broadband scores in the in the internalising, externalising and dysregulation classes.

**
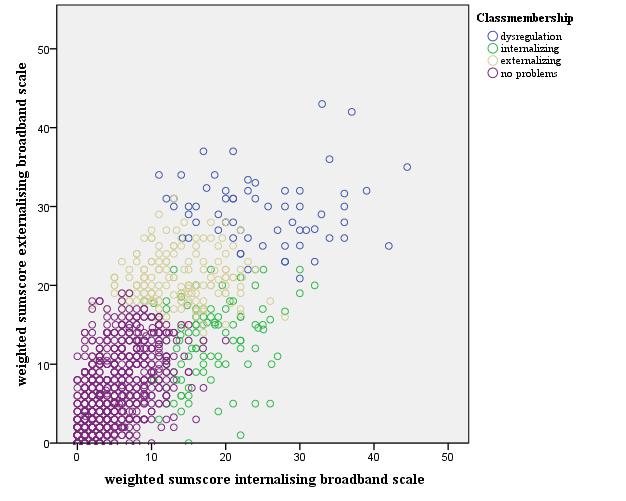
**

**Supplementary Fig. 3** Mean T-scores of dysregulation class, the internalising class and the externalising class

*Note.* T-scores around 65 and higher were considered high, T-scores around 60 were considered moderate (in line with mean T-scores of 57–62 that were found for children referred to a mental health institution [[1](#_ENREF_1)] and T-scores around 55 were considered to be mild problem scores. In comparison, a matched group of non-referred children from the general population had mean T-scores of 54 [[1](#_ENREF_1)].

**Supplementary Fig. 4**

NEPSY-II-NL performance in the internalising, externalising, and dysregulation classes in a sample excluding children with probable ASD (n=978)


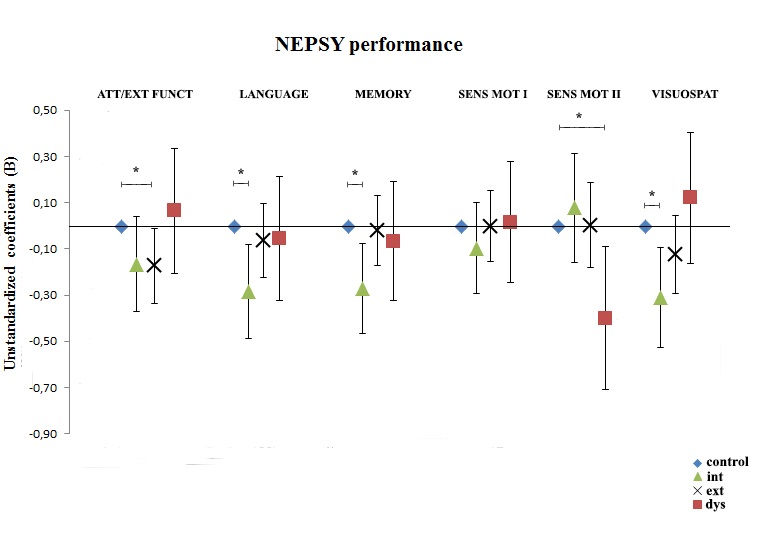


*Note.* The no problems class (n=741) is the reference. Externalising (n=125), internalising (n=76), dysregulation (n=36). Model was adjusted for age and gender. ^*^p<.05. Error bars represent 95% confidence intervals of the regression coefficients. The no problems class is the reference and has no error bars.

| **Supplementary Table 1.** Spearman correlations NEPSY-II-NL subtestscores and corresponding domainscore (*n*=1,307) | | | | | | | | | |
| --- | --- | --- | --- | --- | --- | --- | --- | --- | --- |
|  | |  | **Attention and Executive Functioning domain score** | **Language domain score** | **Memory and Learning domain score** | **Sensorimotor Functioning I: speed accuracy interaction score** | **Sensorimotor Functioning II: pencillifts** | **Visuospatial Processing domain score** |  |
| Auditory Attention | | |  |  |  |  |  |  |  |
|  | Total score | | 0.61^**^ |  |  |  |  |  |  |
|  | Commission errors | | -0.42^**^ |  |  |  |  |  |  |
|  | Omission errors | | -0.61^**^ |  |  |  |  |  |  |
|  | Inhibition errors | | -0.29^**^ |  |  |  |  |  |  |
| Response Set | | |  |  |  |  |  |  |  |
|  | Total score | | 0.80^**^ |  |  |  |  |  |  |
|  | Commission errors | | -0.68^**^ |  |  |  |  |  |  |
|  | Omission errors | | -0.80^**^ |  |  |  |  |  |  |
|  | Inhibition errors | | -0.53^**^ |  |  |  |  |  |  |
| Statue | | |  |  |  |  |  |  |  |
|  | Total score | | 0.48^**^ |  |  |  |  |  |  |
|  | Total movements | | -0.42^**^ |  |  |  |  |  |  |
|  | Total sounds | | -0.32^**^ |  |  |  |  |  |  |
|  | Total eye openings | | -0.41^**^ |  |  |  |  |  |  |
| Word Generation | | |  |  |  |  |  |  |  |
|  | Total of correct words Animals | |  | 0.85^**^ |  |  |  |  |  |
|  | Total of correct words Foods/Drinks | |  | 0.87^**^ |  |  |  |  |  |
| Memory for Faces | | |  |  |  |  |  |  |  |
|  | Total score | |  |  | 0.52^**^ |  |  |  |  |
| Memory for Faces – delayed | | |  |  |  |  |  |  |  |
|  | Total score | |  |  | 0.50^**^ |  |  |  |  |
| Narrative Memory | | |  |  |  |  |  |  |  |
|  | Total score free and cued recall | |  |  | 0.82^**^ |  |  |  |  |
|  | Total score free recall | |  |  | 0.84^**^ |  |  |  |  |
|  | Total score recognition | |  |  | 0.56^**^ |  |  |  |  |
| Visuomotor Precision | | |  |  |  |  |  |  |  |
|  | Total speed accuracy interaction score | |  |  |  | -0.96^**^ |  |  |  |
|  | Total pencillifts | |  |  |  |  | -0.99^**^ |  |  |
| Arrows | | |  |  |  |  |  |  |  |
|  | Total score | |  |  |  |  |  | 0.83^**^ |  |
| Geometric Puzzles | | |  |  |  |  |  |  |  |
|  | Total score | |  |  |  |  |  | 0.63^**^ |  |
| Route Finding | | |  |  |  |  |  |  |  |
|  | Total score | |  |  |  |  |  | 0.75^**^ |  |
| *Note.* NEPSY-II-NL = neuropsychological assessment. ^**^ *p*<0.01 | | | | | | | | | |

| **Supplementary Table 2.** | |  |  |  |
| --- | --- | --- | --- | --- |
| Associations between internalising problems, externalising problems and performance on domains of the NEPSY-II NL | | | | |
| **INTERNALISING**, square root transformed | | |  |  |
|  |  | **B (95% CI)** | **β** | ***p*** |
| **Outcomes,** SD score | |  |  |  |
| Attention and executive functioning | Age and gender | -0.08 (-0.12; -0.04) | -0.11 | **<.001** |
|  | Fully adjusted | -0.07 (-0.12; -0.03) | -0.10 | **.001** |
|  |  |  |  |  |
| Language | Age and gender | -0.10 (-0.14; -0.06) | -0.13 | **<.001** |
|  | Fully adjusted | -0.06 (-0.10; -0.03) | -0.09 | **.001** |
|  |  |  |  |  |
| Memory and Learning | Age and gender | -0.08 (-0.12; -0.05) | -0.11 | **<.001** |
|  | Fully adjusted | -0.07 (-0.11-; -0.03) | -0.09 | **<.001** |
|  |  |  |  |  |
| Sensorimotor Primary | Age and gender | -0.04 (-0.08; 0.00) | -0.05 | **.042** |
|  | Fully adjusted | -0.03 (-0.08; 0.01) | -0.04 | .132 |
|  |  |  |  |  |
| Sensorimotor Secondary | Age and gender | -0.02 (-0.06; 0.03) | -0.02 | .412 |
|  | Fully adjusted | -0.01 (-0.07; 0.04) | -0.02 | .544 |
|  |  |  |  |  |
| Visuospatial | Age and gender | -0.07 (-0.11; -0.03) | -0.09 | **.001** |
|  | Fully adjusted | -0.02 (-0.07; 0.03) | -0.03 | .322 |
| **EXTERNALISING**, square root transformed | |  |  |  |
|  |  | **B (99% CI)** | **β** | ***p*** |
| **Outcomes,** SD score | |  |  |  |
| Attention and executive functioning | Age and gender | -0.08 (-0.12; -0.04) | -0.11 | **<.001** |
|  | Fully adjusted | -0.07 (-0.11; -0.03) | -0.10 | **<.001** |
|  |  |  |  |  |
| Language | Age and gender | -0.05 (-0.08; -0.01) | -0.07 | **.009** |
|  | Fully adjusted | -0.02 (-0.05; 0.02) | -0.03 | .332 |
|  |  |  |  |  |
| Memory and Learning | Age and gender | -0.03 (-0.07; 0.01) | -0.04 | .102 |
|  | Fully adjusted | -0.02 (-0.05; 0.02) | -0.02 | .435 |
|  |  |  |  |  |
| Sensorimotor Primary | Age and gender | -0.04 (-0.08; -0.01) | -0.06 | **.023** |
|  | Fully adjusted | -0.03 (-0.07; 0.02) | -0.05 | .066 |
|  |  |  |  |  |
| Sensorimotor Secondary | Age and gender | -0.05 (-0.09; -0.01) | -0.07 | **.014** |
|  | Fully adjusted | -0.05 (-0.09; -0.01) | -0.07 | **.018** |
|  |  |  |  |  |
| Visuospatial | Age and gender | -0.06 (-0.10; -0.02) | -0.08 | **.003** |
|  | Fully adjusted | -0.02 (-0.07; 0.03) | -0.03 | .315 |

*Note.* The first model was adjusted for age at NEPSY-II-NL, age at CBCL and gender. The second model was additionally adjusted for ethnicity, household income, drinking during pregnancy and smoking during pregnancy. β indicates the standardized change in performance per unit change in the square root transformed CBCL broadband score.

*Non-response analysis*

A non-response analysis comparing subjects that were excluded because of missing information on class (n=130) with the study sample (n=1,177) showed that excluded children were more likely to be of non-Dutch origin (**χ**^2^(2,*N*=1,307)=13.61,*p*=.001), had lower IQ (mean difference=-5.35;*t*(1,180)=3.91,*p*<.0001), lower family income (**χ**^2^(2,*N*=1132)=8.38,*p*=.015), and had mothers with lower education levels (**χ**^2^(2, *N*=1188)=8.02,*p*=.018).

transformed CBCL broadband score. Significant p-values are shown in bold.

**Reference**

1. Achenbach TMR, L.A. (2000) Manual for the ASEBA Preschool Forms & Profiles. University of Vermont, Reseach Center for Children, Youth and Families, Burlington, VT.
